# Dynamic ionic radius of alkali metal ions in aqueous solution: a pulsed-field gradient NMR study†

**DOI:** 10.1039/d1ra02301b

**Published:** 2021-06-07

**Authors:** Kikuko Hayamizu, Yusuke Chiba, Tomoyuki Haishi

**Affiliations:** Institute of Applied Physics, Tsukuba University Tennodai Tsukuba 305-8573 Japan hayamizu.k3@gmail.com; Graduate School of Pure and Applied Sciences and Tsukuba Research Center for Energy, Materials Science (TREMS), University of Tsukuba Tennodai Tsukuba 305-8573 Japan; MRTechnology TCI-B5, Sengen Tsukuba 305-0047 Japan

## Abstract

The dynamic behavior of alkali metal ions, Li^+^, Na^+^, K^+^, Rb^+^ and Cs^+^ in aqueous solutions is one of the most important topics in solution chemistry. Since these alkali metals contain nuclear magnetic resonance (NMR) active nuclei, it is possible to directly measure the diffusion constants of the alkali metal ions using the pulsed field gradient (PFG) NMR method. In this paper, the ^7^Li, ^23^Na, ^87^Rb, ^133^Cs and ^1^H resonances are observed for diffusion constants in aqueous solution and the solvent H_2_O. Until now, the values of the diffusion constant have been lacking when discussing hydration effects around alkali metal ions. It is known that the static ionic radius (*R*_ion_) increases with increasing the atomic number, and the experimental diffusion constants also increase with increasing the atomic number, which is opposite to the Stokes–Einstein (SE) relation. It suggests that alkali metal ions diffuse through a space of 10^−6^ m accompanying the hydrated spheres with a time interval of 10^−3^ s. For each alkali metal ion, the dynamic ionic radius is evaluated.

## Abstract 2

Stokes radius (dynamic ionic radius) of the alkali metal ions *versus* the ionic radius (*R*_ion_) at 303 K. The dotted line is a guide for the 1 : 1 relation.

## Graphical abstract

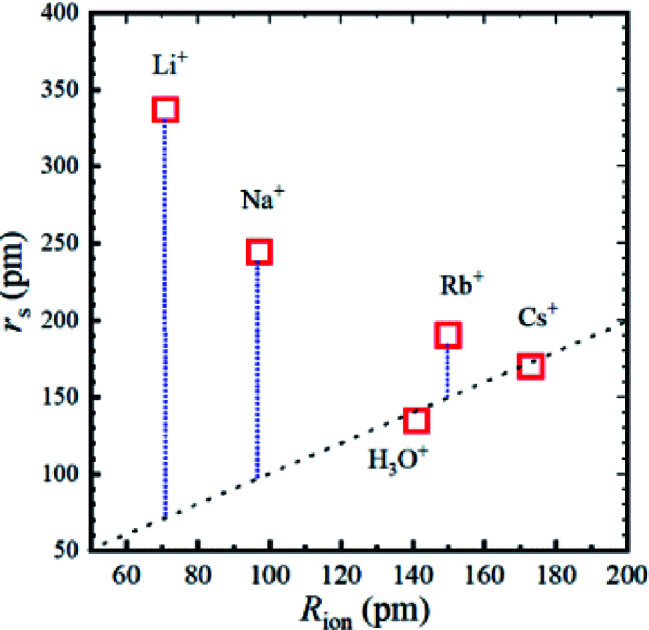

## Introduction

The behavior of alkaline salts in aqueous solutions is an important topic in solution chemistry. Hydrated ions in aqueous solutions have been the subject of numerous studies and fundamental studies have been reviewed.^1,2^ Various techniques have been continuously used for studies on the hydration of the alkali metal ions, for example, in recent papers, neutron diffraction,^3^ large-angle X-ray scattering and double-reference infrared (IR) spectroscopy,^4^ oxygen K-edge X-ray absorption spectroscopy,^5^ and theoretical approaches.^6–8^ Most studies are concerned with the short-range effects of the hydration of alkali metal ions. Because these alkali metals include nuclear magnetic resonance (NMR) active nuclei, the diffusion constants could be measured directly using the pulsed-field gradient (PFG) NMR method. In this paper, the ^7^Li, ^23^Na, ^87^Rb, ^133^Cs and ^1^H resonances were observed for the diffusion constants of the alkali metal ions and the solvent H_2_O. The diffusion measurements were performed in a time scale of 10^−3^ s and were very long compared with the X-ray, IR, and neutron diffraction spectroscopy, and belong to macroscopic properties such as viscosity and ionic conductivity. The PFG-NMR method measures positional migration over a space of 10^−6^ m. Although measurements of viscosity and ionic conductivity have been widely conducted, experimental values of diffusion constants are limited.

The relationship between the diffusion constant *D* and the viscosity (*η*) is known as the Stokes–Einstein (SE) equation:1
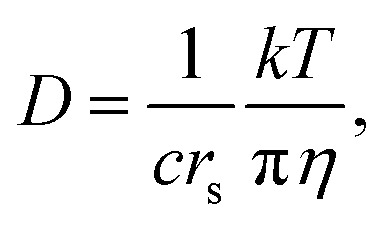
where *k* is the Boltzmann constant, *r*_s_ is the Stokes radius of the diffusing species, and *c* is the constant, for which the theoretical ranges are between 4 and 6 for slip and stick boundary conditions, respectively.^9^

The PFG-NMR method can afford directly the diffusion constants of NMR active nuclei. As *D* is related to the ionic conductivity *via* the Nernst–Einstein relation, the experimental *D* values of Li solution electrolytes for Li-ion batteries (LIB) have been piled up for Li^+^(^7^Li) and anions (^19^F, ^11^B, and others) in organic solvents.^10^

Ionic liquids are another class of solution electrolyte and we have reported temperature-dependent physical constants such as density, ionic conductivity, *η*, and *D* for various ionic liquids and Li salt-doped ionic liquids. *D* is macroscopic in nature, and each electrolyte component has a different value, which can be measured independently for an individual nucleus. Experimental values of *D* relate well with the experimental *η* by the SE relation. The plots of *D versus kT*/πη are always linear and the gradients afford the experimental values of 1/*cr*_s_. Discussion on the constant *c* and the Stokes radius *r*_s_ is necessary for practical systems. Reasonable *r*_s_ values may be calculated by molecular orbital methods, and the *c* values were evaluated experimentally in ionic liquid systems. We have reported the experimental *c* values in the SE relation for the cations, anions and solvated Li^+^ in ionic liquids composed of cations, 1-ethyl-3-methylimidazolium (EMIm) and *N*-methyl-*N*-propyl-pyrrolidinium (P_13_).^11,12^ The anions were bis(trifluoromethanesulfonyl)amide (TFSA) and bis(fluorosulfonyl)amide (FSA) and the systems were prepared without and with doping of a Li salt. Using the temperature-dependent values of *D* and *η*, the plots of *D versus k*/π*η* were always linear. From the gradient of 1/*cr*_s_, experimental value of *cr*_s_ was evaluated for each component. The *c* value was estimated assuming that the *r*_s_ value is the van del Waals radius of the molecular orbital calculation. The approximate *c* values were 2.4 to 3.1 for cations (EMIm and P_13_), 2.7 to 3.7 for anions (TFSA and FSA), and 3.5 to 4.5 for the Li^+^ solvated by anions. In the ionic liquid systems, the *c* values of the cation are smaller than those of the anion. It indicates that the experimental measurable values *D* and *η* are well explained by the SE relation, and the *cr*_s_ value is a constant in each system. It is important to note that *c* is not a universal constant.

In recent years, alkali metals and alkaline earth metals such as Na^+^, K^+^, Mg^2+^, and Ca^2+^ have been targeted for next-generation energy storage systems from the viewpoint of resource conservation. To date, organic solvents have been widely used in LIBs. The possibility of using aqueous solutions is being explored for the prevention of volatility, in addition of the usages of polymer and solid electrolytes. Similarly, fuel cell technology is important in its contribution to power sources. In 1988, diffusion coefficients of Li^+^, Na^+^, and Cs^+^ were observed using steady-state field gradient spin-echo NMR method in aqueous solutions.^13^ Recently, ^1^H^+^, ^7^Li^+^, ^23^Na^+^ and ^133^Cs^+^ diffusion constants were measured in cation-exchange membranes by the PFG-NMR method.^14^ Furthermore, Na^+^ and K^+^ ions are important in biological systems. In humans, Na^+^ is present outside the cells and K^+^ inside the cells. In particular, Na^+^ circulates mainly in the form of blood, and its concentration is maintained by the kidneys. Na^+^ is the main factor in the formation of osmotic pressure to retain water.

We observed the diffusion constants of ^7^Li^+^, ^23^Na^+^, ^87^Rb^+^ and ^133^Cs^+^ in aqueous solutions. Using the reported values of *D* and *η* of pure H_2_O, the *c* value was estimated to be 4.8. From the experimental diffusion constants of alkali metal ions and solvent H_2_O, the dynamic radius of each alkali metal ion is proposed. The large hydration ability of Li and Na ions is discussed.

## Experimental

The PFG NMR measurements were performed at 303 K by using a Tecmag Apollo and an NT-NMR console (Houston, TX) equipped with a JEOL PFG-probe with a well-shaped rectangular PFG of the maximum strength is 24 Tm^−1^ (Tokyo). The basic properties of each nuclei are summarized in Table 1, where the *T*_1_ and *T*_2_ values of the alkali metal ions were obtained in the diluted aqueous solutions at 303 K in this study. The *T*_1_ and *T*_2_ values are important to determine the diffusion measuring conditions. The PFG NMR pulse sequences are shown for the Hahn echo pulse sequence (^1^H, ^7^Li, ^23^Na, and ^133^Ce) and the stimulated echo pulse sequence (^87^Rb) in Fig. S1 in ESI.† The *T*_1_ and *T*_2_ values at 303 K are sufficiently long for ^1^H, ^7^Li, ^23^Na, and ^133^Cs to use the Hahn-echo PFG NMR pulse sequence and the measurements were performed with the PFG strength 1.26 Tm^−1^. The precise measurement details are described in the ESI.† Because the *T*_1_ and *T*_2_ values of ^87^Rb^+^ were extremely short, the stimulated echo PFG pulse sequence was used with the *g* value of 10.0 Tm^−1^ (Fig. S2, ESI†). ^39^K belongs to a low-γ nuclei and a JEOL ECZ-400 spectrometer can measure the ^39^K spectra at 18.6 MHz. The *T*_1_ and *T*_2_ values for ^39^K were short (Table 1). Unfortunately, the maximum PFG strength equipped was 0.3 Tm^−1^. Several attempts were made to obtain the ^39^K diffusion constant for the saturated aqueous solution (3 M), but a reliable *D* value for K^+^ could not be obtained because of poor decay of the echo attenuation. The repetition time of every experiment including relaxation time measurement was set at least five times longer than *T*_1_

**Table 1:** Basic properties and *T*_1_ and *T*_2_ at 303 K for the nuclei of alkali metal ions

Nucleus	*T*_1_	*T*_2_	Frequency (MHz)	I	*Q* b (10^−31^ m^2^)
^7^Li	25 s	5 s	78.45	3/2	−40.1
^23^Na	46 ms	23 ms	53.39	3/2	104
^39^K	64 ms	32 ms	18.68^a^	3/2	58.5
^87^Rb	1.8 ms	1.2 ms	27.85	3/2	133.5
^133^Cs	9 s	1.2 s	26.47	7/2	−3.43
^1^H (H_2_O)	3.5 s	—	201.85	1/2	—

a Observed by a JEOL ECZ-400 spectrometer at the field strength of 9.404 T.

b Nuclear quadrupole moment from Bruker NMR properties of selected isotopes.

The aqueous sample was placed in a 5 mm NMR microtube (BMS-005J, Shigemi, Tokyo) to a height of 5 mm. The aqueous LiCl and NaCl samples were prepared by diluting concentrated samples.

## Results and discussion

### Concentration dependences of diffusion constants for Li^+^, Na^+^ and solvent H_2_O

**Fig. 1:**
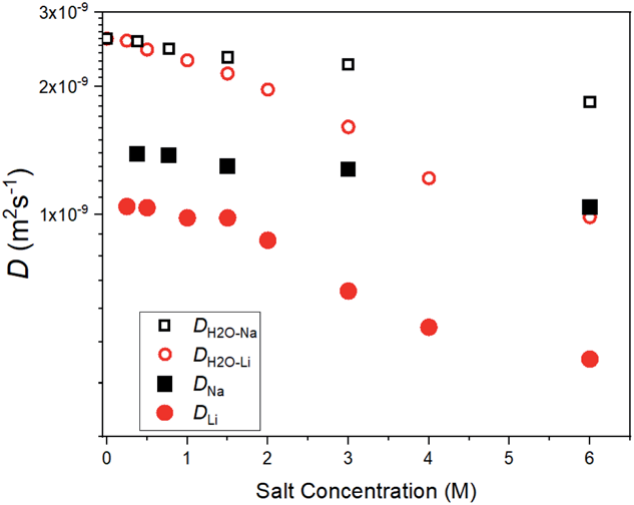
Concentration-dependent diffusion constants for ^7^Li (*D*_Li_), ^23^Na (*D*_Na_), and ^1^H (solvent H_2_O, *D*_H_2_O–Li_ and *D*_H_2_O–Na_) at 303 K in aqueous solutions.

The salt concentration dependences of the diffusion constants for ^7^Li (*D*_Li_), ^23^Na (*D*_Na_), and ^1^H solvent H_2_O (*D*_H_2_O–Li_ and *D*_H_2_O–Na_) are shown in Fig. 1. In homogenous solutions, a unique diffusion constant (*D*) can be determined independently of measurement conditions such as the diffusion time (*Δ*). In diluted regions (≤2 M), each diffusion constant was a single component without dependence of *Δ* (10^−3^ s order). The obtained *D* values are physical constants because of independence of the measuring parameters of PFG-NMR. In general, for heterogeneous materials such as zeolites and solid electrolytes, the apparent diffusion constant may depend on *Δ* and *g*, in which case the diffusion constant as a physical constant cannot be obtained. In very concentrated regions (≥4 M), a small amount of a slower component coexists in *D*_H_2_O_, *D*_Li_ and *D*_Na_, and strictly speaking, they are not homogeneous systems; this requires further study.

The *D*_Li_ values were smaller than the *D*_Na_ values over the whole concentration range. A larger concentration dependence was observed for *D*_Li_ than for *D*_Na_. Solvent H_2_O diffusion constants decreased slightly with an increase in concentration and the concentration dependence of *D*_H_2_O–Li_ was larger than that of *D*_H_2_O–Na_, correlating with the relations of the *D*_Li_ and *D*_Na_. The ratios of *D*_H_2_O–Li_/*D*_Li_ and *D*_H_2_O–Na_/*D*_Na_ were plotted against the salt concentration in Fig. S4 in the ESI† and they were almost constant for the concentration and *D*_H_2_O–Li_/*D*_Li_ > *D*_H_2_O–Na_/*D*_Na_. The temperature-dependent diffusion constants of pure H_2_O have been reported^15^ and the value of 2.6 × 10^−9^ m^2^ s^−1^ at 303 K is consistent with the present value for H_2_O without including salt.

We have reported the salt concentration dependences of the diffusion constants of Li^+^ ions for LiBF_4_ and LiN(SO_2_CF_3_)_2_ in two organic solvents, propylene carbonate (PC) and γ-butyrolactone (GBL). Near the infinitesimal concentration region, clear concentration dependences of the diffusion constants of Li ions and anions were observed.^16^ In the aqueous solutions, the concentration dependence of *D*_Li_ is small compared with those in organic solvents.

In liquid crystal systems, the SE equation (eqn (1)) holds well between experimental *D* and *η* as indicated.^11,12^ In many cases, the constant *c* has been treated as 6, but it varies widely for each species in ionic liquids. In this study, we tried to find the *c* value for H_2_O. In the ESI,† using published data of the temperature-dependent *D*_H_2_O_ and *η* of H_2_O, the plot of the SE relation (*D versus* 1/π*η*) is linear with the gradient 1/*cr*_s_. The experimental value of *cr*_s_ is 673 pm. When the ionic radius *R*_ion_ of H_3_O^+^ (141 pm) is assumed to be *r*_s_, the *c* value is 4.8 for H_2_O. Following the classical relation, *c* value is either 4 nor 6, then the *r*_s_ values are 168 or 112 pm for 4 or 6, respectively. The estimate of *c* = 4.8 of H_2_O is tentative, but we will use this value in our discussion. The viscosity of the solution at each salt concentration was estimated by eqn (2).2
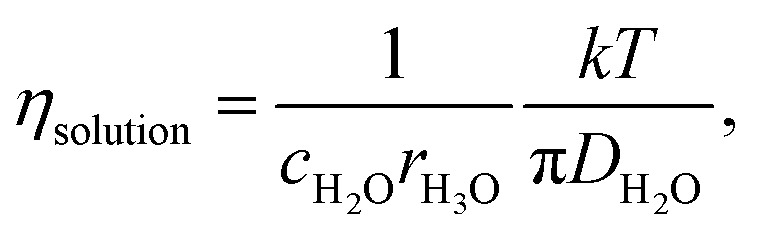


**Fig. 2:**
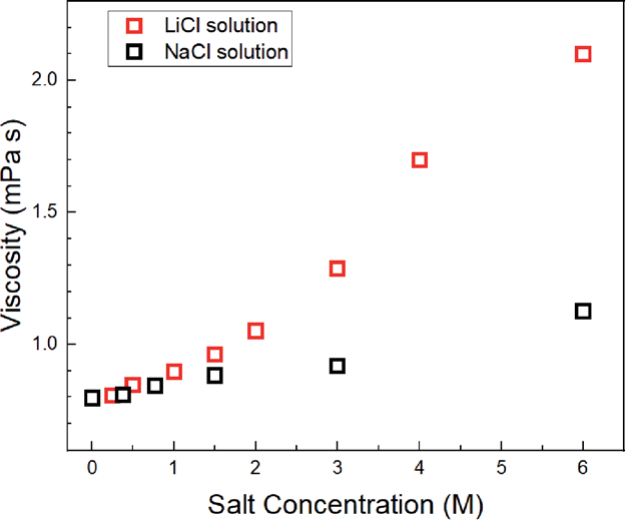
Estimated viscosity for the aqueous LiCl and NaCl solutions at 303 K.

Assuming *c* = 4.8 and *r*_H_3_O_ = 141 pm, the concentration-dependent viscosity was estimated from the *D*_H_2_O_ for the aqueous LiCl and NaCl solutions (Fig. 2).

The increase in viscosity with the concentration increase in aqueous LiCl solutions was larger than that in the aqueous NaCl solutions, which were calculated by *D*_H_2_O–Na_ and *D*_H_2_O–Li_ (Fig. 1).

The dynamic Stokes radius for the alkali metal ions (Li and Na) is estimated at each concentration of aqueous solution with *c* = 4.8:3
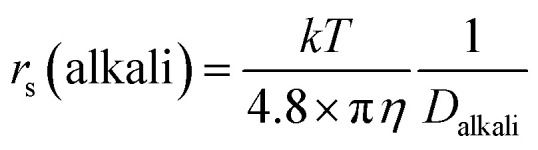


**Fig. 3:**
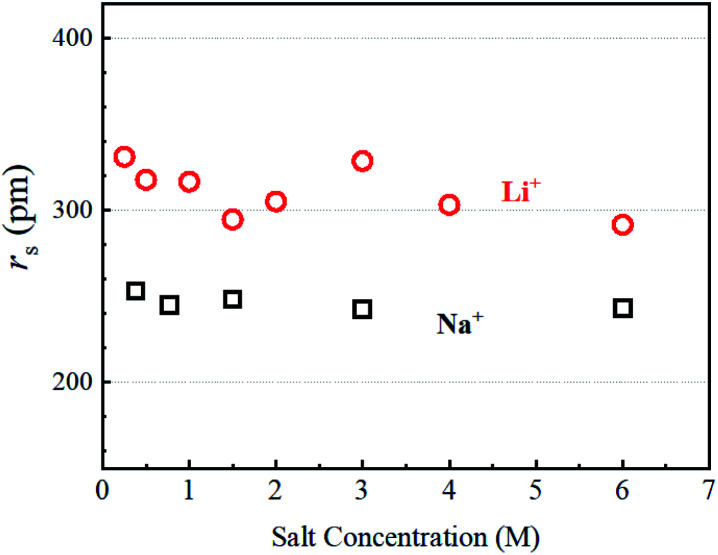
Estimated Stokes radius, *r*_s_, of Li^+^ and Na^+^ ions *versus* the salt concentration.

The estimated values are plotted *versus* the salt concentration for the LiCl and NaCl solutions in Fig. 3.

It is not surprising that Fig. 3 is similar to Fig. S4,† but Fig. 3 has a clearer meaning of the *Y*-axis. The estimated *r*_s_(Li^+^) values scattered slightly without an explicit trend *versus* the salt concentration. The *r*_s_(Na^+^) values were almost independent of salt concentration. In this stage, the ion association effects must be considered. It is known that ion association increases with increase in the salt concentration measured in the ionic conductivity. We reported the ion association effects in organic Li^+^ solutions, where the solvation around Li^+^ is insensitive to the ion association.^17^ Because of the strong solvation ability of the organic solvent with high dielectric constant such as PC and GBL, the solvated Li^+^ ions associate with anions for ion pairing. Organic anions are weakly solvated in organic solution electrolytes. The ion association in organic solvents takes place between solvated Li^+^ and solvated anions.^16^ In aqueous solutions, the ion association effects on the hydration can be assumed to be negligible in diluted regions. The *r*_s_(Li) and *r*_s_(Na) values suggest that the dynamic diffusion constants include the H_2_O solvation effects. The hydration around Li^+^ is larger than that around Na^+^ and the hydration effect is insensitive to salt concentration at 303 K.

It is noted that in aqueous solutions, ^7^Li *T*_1_ values varied from 25 to 8 s with the increase in salt concentration and they were very longer than the ^7^Li *T*_1_ values in organic solvents (under 3 s) and in ionic liquids (∼0.3 s).^11,12^ The relaxation mechanism of ^7^Li *T*_1_ is mainly quadrupole interaction, and in the extreme narrowing condition, 1/*T*_1_(^7^Li) is proportional to quadrupole coupling constant (*e*^2^*qQ*/*h*)^2^, where *q* is the electric field gradient around ^7^Li nuclei and deeply related to the shape of circumstance of ^7^Li nuclei. The better cubic shape around the Li^+^ ion reduces *q*, and *T*_1_(^7^Li) value becomes longer because of the smaller quadrupole effect. The longer ^7^Li *T*_1_ values in the diluted aqueous solutions suggest a good cubic symmetry of ^7^Li. The ^23^Na *T*_1_ values were shorter owing to the larger nuclear quadrupole moment (Table 1) and varied from 64 to 46 ms with the increase in salt concentration. ^1^H *T*_1_ (H_2_O) varied from 3.6 to 2.1 s (Li solution) and from 3.6 to 3.1 s (Na solution.) with the increase in salt concentration. The larger degree of the decrease in ^7^Li *T*_1_ and H_2_O *T*_1_ suggests stronger interactions in Li–H_2_O compared with that in Na–H_2_O in aqueous solutions. The decrease of ^7^Li *T*_1_ with the increase of the salt concentration suggests decreasing the cubic symmetry of the solvation structure around Li^+^ and Na^+^.

### Diffusion constants of alkali metal ions and solvent H_2_O

In diluted regions, the values of *D*_Li_, *D*_Na_ and *D*_H2O_ are shown to be insensitive to the salt concentration and the data about 1 M are used to compare the data of other alkali metal ions of Rb^+^ and Cs^+^. The salts used in this study were Rb_2_CO_3_ and Cs_2_CO_3_. The concentration of aqueous solutions was about 1 M. It was confirmed that with dilution, the *D*_Rb_*D*_Cs_ and *D*_H2O_ values did not vary within experimental error. Attempts to obtain the ^39^K diffusion constants were made using a JEOL ECZ-400 spectrometer under small PFG strength (0.3 Tm^−1^). Unfortunately, a reliable value of the *D*_K_ was not observed. The data of ^39^K *T*_1_ and *T*_2_ obtained under the field strength of 9.404 T (^1^H frequency 400.39 MHz) are relatively short (Table 1).

**Fig. 4:**
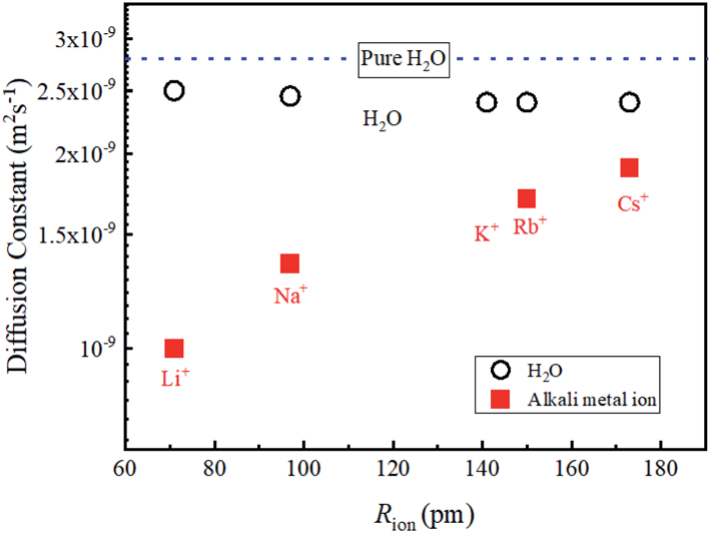
Diffusion constants of alkali metal ions about 1 M in aqueous solution at 303 K *versus* the ionic radius *R*_ion_. The *D*_H_2_O_ values (including diluted KCl solution) are shown and the *D*_H_2_O_ value of pure H_2_O is slightly faster than those of the solvent H_2_O.

The diffusion constants of alkali metal ions and solvent H_2_O about 1 M including a diluted KCl solution at 303 K are plotted *versus* the reported ionic radius (*R*_ion_)^2^ in Fig. 4.

The *D*_Li_ is the slowest and increases for Na^+^, Rb^+^, and Cs^+^ (Fig. 4), and the ionic radius of Li (*R*_ion_(Li)) is smallest of the alkali metal ions. Faster *D* is observed for alkaline metal ion with larger *R*_ion_. This trend is opposite to that of *D* which is proportional to 1/*R*_ion_

The diffusion constants of solvent H_2_O are slightly slower than the *D* value of pure H_2_O. This suggests that the viscosity of the alkali ion aqueous solutions changes little in diluted regions. To obtain the Stokes radius (*r*_s_) (*i.e.*, dynamic ionic radius), the viscosity of each solution was calculated (eqn (2)) and then the *r*_s_ was evaluated (eqn (3)). Here we assume that the constant *c* = 4.8 is the same as that of pure H_2_O. To estimate dynamic ionic radius the measurements of *D*_metal_ and *D*_H_2_O_ of the same sample were performed. The viscosity of the solution was estimated from the solvent *D*_H_2_O_ as 
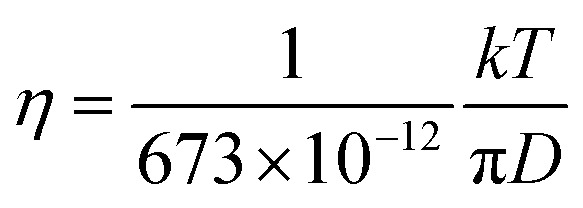
 following to the SE relation (experimental value of *cr*_s_ of pure H_2_O is 673 pm). Then the dynamic ionic radius *r*_s_ of an alkali metal was evaluated from the *D* of an alkali metal as 
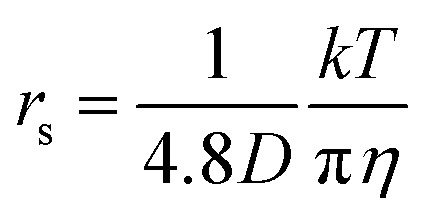
, where *c* is assumed 4.8.

The diffusion constants of Li^+^, Na^+^, Cs^+^ were reported in D_2_O at 298 K for 1 M concentration to be 0.92, 1.2, and 1.8 × 10^−9^ m^2^ s^−1^, respectively.^13^ Our values are consistent to the reported data, and the trend that the increase of the *D* values as the increase of the alkali metal size is quite consistent with each other.

The *r*_s_ values were obtained experimentally on timescale of 10^−6^ s which is much longer than the commonly used timescale for spectroscopy and MO calculations. This means that PFG-NMR experiments can provide important information for electrochemistry in the similar timescale of ionic conductivity and viscosity.

**Fig. 5:**
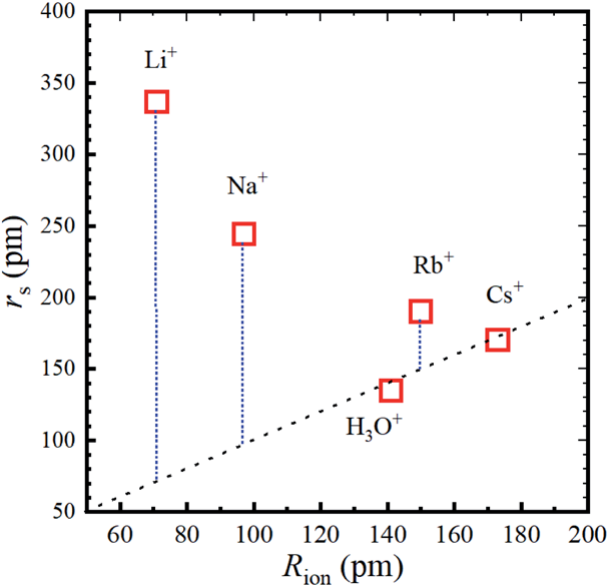
Stokes radius (*i.e.*, dynamic ionic radius) of the alkali metal ions *versus* the ionic radius (*R*_ion_) at 303 K. The dotted line is a guide for the 1 : 1 relation.

The calculated *r*_s_ values are plotted *versus R*_ion_ in Fig. 5 and the numerical values are given in Table 2.

**Table 2:** Experimental values of *D*_alkali_ and *D*_H_2_O_ in diluted region at 303 K and estimated viscosity, dynamic ionic radius *r*_s_, and radial hydration number

Nucleus	Experimental		Estimated			*R*_ion_ (pm)
	*D*_alkali_ 10^−9^ m^2^ s^−1^	*D*_H_2_O_ 10^−9^ m^2^ s^−1^	*η* (mPa s)	*r*_s_ (pm)	Radial hydration^b^	
Li^+^	1.0	2.5	0.829	337	1.9	71
Na^+^	1.35	2.4_5_	0.846	244	1.0	97
K^+^		2.4	0.864	—	—	141
Rb^+^	1.7	2.4	0.864	190	0.28	150
Cs^+^	1.9	2.4	0.864	170	0	173
Pure H_2_O		2.6	0.7972^a^			141

a https://wiki.anton-paar.com/jp-jp/water/, accessed January15, 2021.

b Radial hydration number = (*r*_s_ − *R*_ion_)/*R*_H_2_O_.

**Fig. 6:**
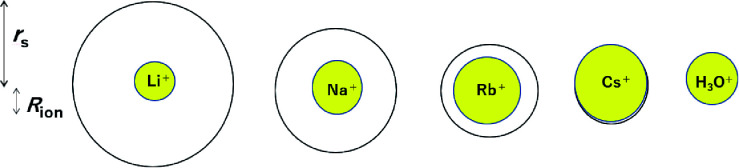
Relations between the dynamic ionic radius (*r*_s_) and static ionic radius (*R*_ion_) for the alkali metal ions in aqueous solutions.

For a better understanding the relations between the dynamic and static ionic radii, Fig. 6 illustrates the relations between *r*_s_ and *R*_ion_.

**Fig. 7:**
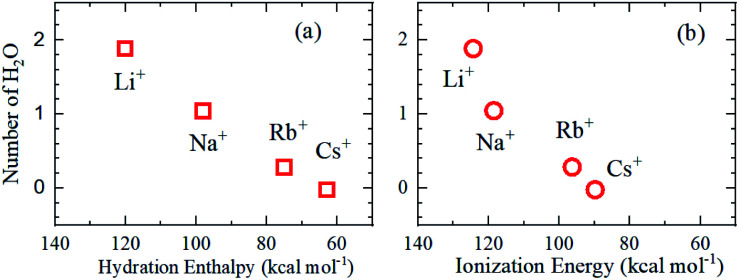
H_2_O number in the radial direction *versus* (a) hydration enthalpy and (b) ionization energy.

As is well known, the larger the atomic number of alkali metal, the larger the *R*_ion_. Experimental results on the dynamic ionic radius show an opposite trend; a reduction in the Stokes radius *r*_s_ with increasing alkali atomic number. The dynamic radius of Li ion was the largest compared with the static ionic radius. To interpret this tendency, it is assumed that the dynamic ionic radius is induced by hydration and the hydrated alkali ions diffuse accompanied by H_2_O molecules. A simple calculation was performed to estimate the number of H_2_O molecules in the radial direction (the hydration number) as (*r*_s_ − *R*_ion_)/*R*_H_2_O_ for each alkali metal ion (Table 2). The hydration number of Li^+^ is about 2 in the radial direction, and larger alkali metal ions are less hydrated. Then the hydration number is plotted *versus* hydration enthalpy and ionization energy reported^18^ in Fig. 7(a) and (b), respectively.

The hydration numbers of the alkali metal ions determined by the PFG NMR method correlate well with the hydration enthalpy. The relationship between hydration enthalpy and ionization energy for numerous ions have been discussed widely,^18^ and in this paper, the plot of the hydration number *versus* ionization energy for the alkali metal ions suggests that the hydration of Li^+^ is very large.

## Conclusions

The diffusion constants of the NMR-active nuclei of alkali metal ions have been determined by the PFG NMR method on a timescale of 10^−3^ s in a space of 10^−6^ m. The initial position of the NMR-active nucleus is marked by the first PFG and after time interval *Δ* (10^−3^ s order), the migrated position is detected by the second PFG. The dynamic ion radius (*i.e.*, the Stokes radius) of each alkali metal ion was determined. The hydrated sphere diffuses together with the alkali metal ion. The hydration sphere is largest for Li^+^, followed by Na^+^, Rb^+^, and Cs^+^, and Cs^+^ has almost no hydration sphere. During the diffusion period, the PFG NMR method does not provide any information on the H_2_O exchange between the hydration sphere and the bulk.

In this study, the theoretically derived SE relation was experimentally proved for the first time in the case of alkali metal ions in aqueous solution. The experimental value of the constant, *c* = 4.8 for pure H_2_O is reasonable in the aqueous solutions.

## Conflicts of interest

There are no conflicts to declare.

## Supplementary Material

RA-011-D1RA02301B-s001
