# Supplementary material for: Dynamic ionic radius of alkali metal ions in aqueous solution: a pulsed-field gradient NMR study
Source: RSC Adv. 2021 Jun 7;11(33):20252–7. doi: 10.1039/d1ra02301b (PMC9033755; doi:10.1039/d1ra02301b)
Supplement: RA-011-D1RA02301B-s001 [file RA-011-D1RA02301B-s001.pdf]

Electronic Supplementary Material (ESI) for RSC Advances.
This journal is © The Royal Society of Chemistry 2021

Electronic Supplementary Information (ESI)

Dynamic Ionic Radii of Alkali Metal Ions in Aqueous Solution: A Pulsed-Field Gradient NMR Study

Kikuko Hayamizu*[a], Yusuke Chiba[b], and Tomoyuki Haishi[c]

[a]Institute of Applied Physics, Tsukuba University, Tennodai, Tsukuba 305-8573, Japan.

[b]Graduate School of Pure and Applied Sciences and Tsukuba Research Center for Energy, Materials Science (TREMS), University of Tsukuba, Tennodai, Tsukuba 305-8573, Japan

[c]MRTechnology, TCI-B5, Sengen, Tsukuba 305-0047, Japan

PFG-NMR pulse sequences

Diffusion constants of NMR-active nuclei can be observed by the pulsed-field gradient (PFG) NMR method. Basically, two equivalent PFGs are applied on the Hahn pulse sequence shown in Figure S1(a). The echo attenuations are observed with varying PFG magnitude. When the spin-spin relaxation time is short, the stimulated echo (STE) pulse sequence is used in Figure S1(b). In this study, well-shaped rectangular PFGs are used.

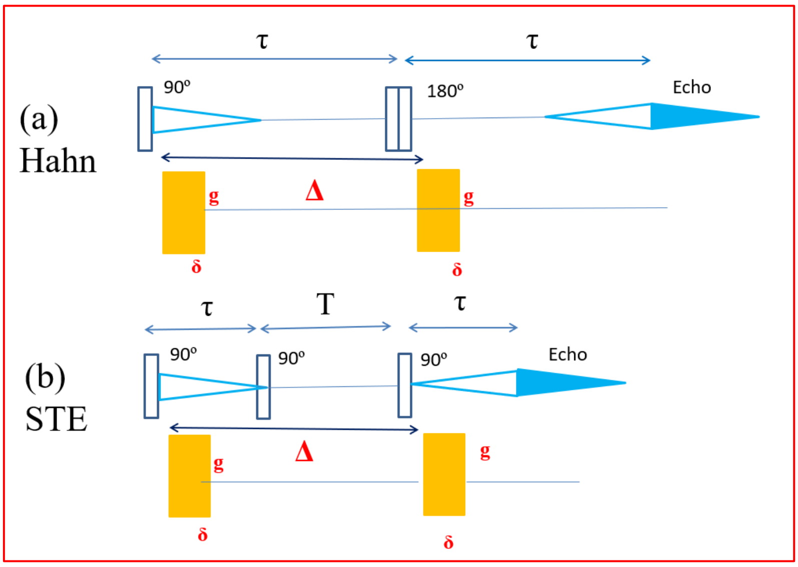

Fig. S1 PFG-NMR pulse sequences for (a) Hahn echo and (b) Stimulated echo.

Two equivalent PFGs with the strength g and duration time δ are applied by the time interval Δ. By varying $g \times \delta$, the echo attenuation decay signals are observed. The echo attenuation S values normalized observed by the smallest $g \times \delta$ are plotted following to the Stejskal and Tanner equation (1)

$$S(g, \delta, \Delta) = \frac{E}{E_o} = \exp\left(-\gamma^2\delta^2 g^2 D\left(\Delta - \frac{\delta}{3}\right)\right) = \exp(-bD) \qquad (1),$$

γ is the gyromagnetic ratio of the observing nucleus and D is the diffusion constant. When the PFG is a rectangle with good shape, the D values agree whether the measurement is made by varying δ with a fixed g or by varying g with a fixed δ.[S1] In heterogenous systems, D values sometimes depend on Δ and g, and g-fixed measurements are reliable.

The measuring conditions of $^{7}Li^{+}$, $^{23}Na^{+}$, and $^{133}Cs^{+}$ in the aqueous solvent are following.

^{7}Li: g = 1.26 Tm^{-1} and δ = 0.2 ~ 2 ms (varied). For various Δ values from 20 to 100 ms, the determined values of D_{Li} agreed within experimental errors in the diluted region (less than 2 M).

^{23}Na: g = 1.26 Tm^{-1} and δ = 0.2 ~ 2 ms (varied). For various Δ values from 20 to 100 ms. The determined value of D_{Na} agreed within experimental errors in the diluted region (less than 2 M).

^{133}Cs: g = 1.26 Tm^{-1}, Δ = 100 ms, δ = 0.2 ~ 2 ms (varied).

Diffusion measurement of ^{87}Rb

Because of extremely short relaxation times of ^{87}Rb (T_1 = 1.8 ms and T_2 = 1.2 ms), the ^{87}Rb diffusion constant was measured by the stimulated pulse sequence with strict measuring conditions in Fig. S2. Eddy current effect disappeared within 0.2 ms after the application of the PFG (10 Tm^{-1}) in the present measuring conditions. The eddy current length was already examined. [S2]

Due to very short T_1 and T_2 of ^{87}Rb due to large nuclear quadrupoler moment (Q) in Table 1, the setting of the PFG measuring parameters is limited to a short Δ value. Because of the uniform Rb^{+} aqueous solution, negligible Δ dependence of D_{Rb} can be assumed.

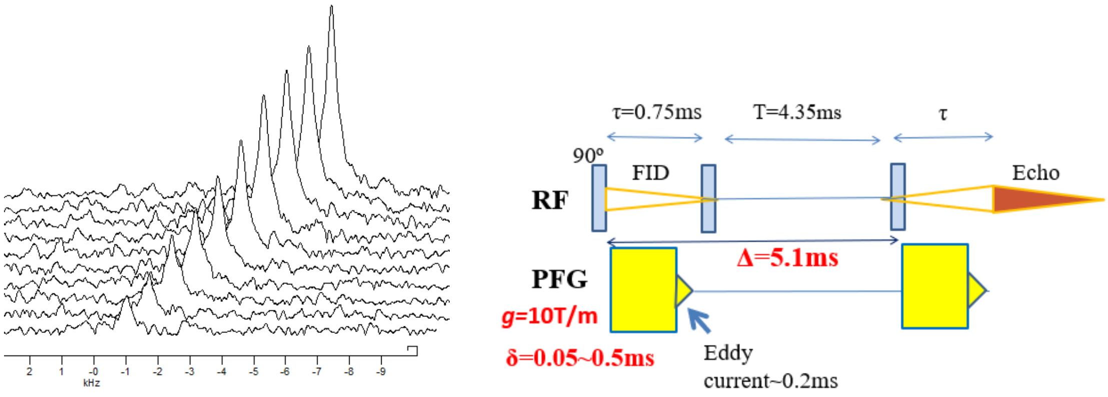

Fig. S2 ^{87}Rb echo attenuation signals measured by the STE pulse sequence shown in the right. The PFG strength, g was 10.0 Tm^{-1}, τ = 0.75 ms (setting of short τ was possible because of fast decay of the eddy current effects, about 0.2 ms), Δ = 5.1 ms, and δ = 0.05 ~ 0.5 ms (varied). An overnight accumulation was performed (about 8,000 accumulations).

Stokes-Einstein relationship for pure H_2O

The relationship between a diffusion constant (D) and viscosity (η) is well established by the Stokes-Einstein (SE) equation (S1) with unestablished constant c (theoretically between 4 and 6) and the Stokes radius r_s for a diffusing particle.

$$D = \frac{1}{c r_S} \frac{kT}{\pi\eta} \qquad (S1)$$

The temperature-dependent experimental values of D[S3] and η[S4] are available for pure H_2O. The D_{H2O} is plotted versus $kT/\pi\eta$ in Fig. S3.

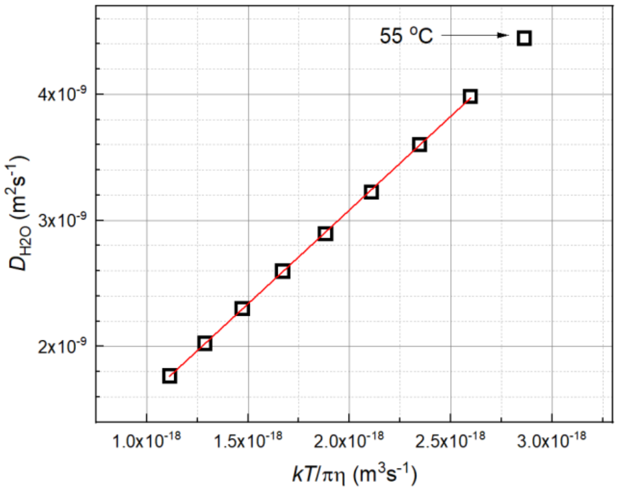

Fig. S3 Experimental D_{H2O} is plotted versus $kT/\pi\eta$.

The good linear relationship between D and $kT/\pi\eta$ was obtained excluding a D_{H2O} data at 55 °C. The deviation of the D value towards larger one is probably the convection effect (compared with our unpublished data measured by using a specified NMR sample tube[S5]). From eqn (S1), the gradient is equal to $1/cr_s$ and the calcurated cr_s is the experimental constant in the SE relation. The experimental value, cr_s = 673 pm. The ionic radius of H_3O^+ (r_s = 141 pm[S6]) suggests the constant c = 4.8 for H_2O in the SE relation, which is between the theoretical values 4 and 6 for the slit and stick boundary conditions. When the c value is assumed to be 4 or 6, the radieus of H_2O becomes 168 or 112 pm, respectively. It is noted that the bond length of O-H in H_2O is 96 pm, which is a base to calculate van del Waals volume of H_2O molecule.

Relations of D_{Li}-$D_{H2O\text{-}Li}$ and D_{Na}-$D_{H2O\text{-}Na}$ against the salt concentration

From the SE relation, the ratio of the diffusion constants is related to the size of diffusing species (r_s) as

$D_{H2O}/D_{Li} \propto r_s(Li)/r_s(H_2O)$

$D_{H2O}/D_{Na} \propto r_s(Na)/r_s(H_2O)$

The ratios were calculated for Li^+ and Na^+ systems and plotted against the salt concentration (Fig. S4).

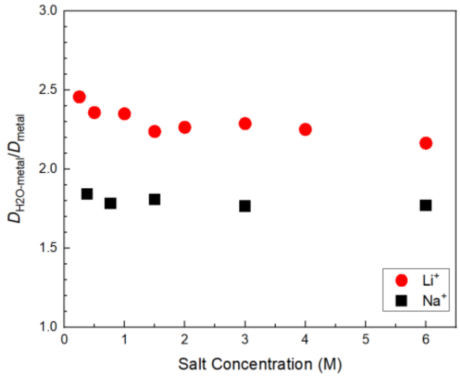

Fig. S4 Ratios of $D_{H2O\text{-}Li}/ D_{Li}$ and $D_{H2O\text{-}Na}/D_{Na}$ are plotted against the salt concentration.

The ratio of Li^+ system changed from 2.4$_5$ to 2.2$_5$ with increasing the salt concentration and that of Na^+ system was almost constant around 1.8. It is clearly shown that Li^+ is more hydrated than Na^+, but it is not certain that in the dilution limit Li^+ is more hydrated and Na^+ is not dependent on the salt concentration in hydration.

References

S1. K. Hayamizu, S. Seki, T. Haishi, Lithium ion micrometer diffusion in a garnet-type cubic $Li_7La_3Zr_2O_{12}$ (LLZO) studied using ^{7}Li NMR spectroscopy. *J. Chem. Phys.*, 2017, **146**, 024701. 10.1063/1.4973827

S2. K. Hayamizu, S. Seki, Long-range Li ion diffusion in NASICON-type $Li_{1.5}Al_{0.5}Ge_{1.5}(PO_4)_3$ (LAGP) studied by ^{7}Li pulsed-gradient spin-echo NMR. *Phys. Chem. Chem. Phys*. 2017, **19**, 23485-23491. 10.1039/c7cp03647g

S3. M. Holz, S. R. Heil, A. Sacco, Temperature-dependent self-diffusion coefficients of water and six selected molecular liquids for calibration in accurate ^{1}H NMR PFG measurements. *Phys. Chem. Chem. Phys.*, 2000, ***2***, 4740-4742. 10.1039/b005319h

S4. https:// wiki.anton-paar.com/jp-jp/water/, accessed January15, 2021.

S5. K. Hayamizu, W. S. Price, A new type of sample tube for reducing convection effects in PGSE-NMR measurements of self-diffusion coefficients of liquid samples. *J. Magn. Reson*. 2004, **167**, 328-333. 10.1016/j.jmr.2004.01.006

S6. Y. Marcus, Ionic radii in aqueous solutions. *Chem. Rev.* 1988, **88**, 1475-1498. 10.1021/cr00090a003
